# Urinary metabolic profiling of asymptomatic acute intermittent porphyria using a rule-mining-based algorithm

**DOI:** 10.1007/s11306-017-1305-9

**Published:** 2017-12-04

**Authors:** Margaux Luck, Caroline Schmitt, Neila Talbi, Laurent Gouya, Cédric Caradeuc, Hervé Puy, Gildas Bertho, Nicolas Pallet

**Affiliations:** 1 INSERM U1147, Centre Universitaire des Saints Pères, Paris, France; 2 Université Paris Descartes, Paris, France; 3 Sorbonne Paris Cité, Paris, France; 4 Institut Hypercube, Paris, France; 5 Centre Francais des Porphyries, Hôpital Louis Mourier, Assistance Publique-Hôpitaux de Paris, Colombes, France; 6 INSERM U1149, CNRS ERL 8252, Center for Research on Inflammation (CRI), Université Paris Diderot, Site Bichat, Sorbonne Paris Cité, Paris, France; 7 Laboratory of Excellence, GR-Ex, Paris, France; 8 UMRS 8601 CNRS, Paris, France; 9 Service de Biochimie, Hôpital Européen Georges Pompidou, Assistance Publique-Hôpitaux de Paris, 20, rue Leblanc, 75015 Paris, France

**Keywords:** ^1^H NMR, Porphyrias, Biomarkers, Subgroup discovery

## Abstract

**Introduction:**

Metabolomic profiling combines Nuclear Magnetic Resonance spectroscopy with supervised statistical analysis that might allow to better understanding the mechanisms of a disease.

**Objectives:**

In this study, the urinary metabolic profiling of individuals with porphyrias was performed to predict different types of disease, and to propose new pathophysiological hypotheses.

**Methods:**

Urine ^1^H-NMR spectra of 73 patients with asymptomatic acute intermittent porphyria (aAIP) and familial or sporadic porphyria cutanea tarda (f/sPCT) were compared using a supervised rule-mining algorithm. NMR spectrum buckets bins, corresponding to rules, were extracted and a logistic regression was trained.

**Results:**

Our rule-mining algorithm generated results were consistent with those obtained using partial least square discriminant analysis (PLS-DA) and the predictive performance of the model was significant. Buckets that were identified by the algorithm corresponded to metabolites involved in glycolysis and energy-conversion pathways, notably acetate, citrate, and pyruvate, which were found in higher concentrations in the urines of aAIP compared with PCT patients. Metabolic profiling did not discriminate sPCT from fPCT patients.

**Conclusion:**

These results suggest that metabolic reprogramming occurs in aAIP individuals, even in the absence of overt symptoms, and supports the relationship that occur between heme synthesis and mitochondrial energetic metabolism.

**Electronic supplementary material:**

The online version of this article (10.1007/s11306-017-1305-9) contains supplementary material, which is available to authorized users.

## Introduction

Metabolic profiling allows the identification of metabolic markers associated with diseases and supports the formulation of new pathophysiological hypothesis. One of the most employed analytical techniques for untargeted metabolomics is proton based nuclear magnetic resonance (^1^H-NMR) spectroscopy (Goodacre et al. 2004; Shulaev 2006). It provides information on molecular structures and on absolute or relative concentrations of compounds in complex mixtures.

Metabolomic studies generate high-dimensional datasets requiring the use of powerful modeling techniques (Sugimoto et al. 2012). The most commonly used modeling techniques for metabolomics profiling are partial least squares (PLS) and its derivate (Goodpaster et al. 2010; Liland 2011; Lindon and Nicholson 2008; Trygg and Wold 2002). However, these techniques do not perform an exhaustive exploration of the data. To overcome this limitation, we used a descriptive and easily understandable approach for metabolic profiling, which is based on a supervised rule-mining algorithm that locally and exhaustively explores the variable space of ^1^H-NMR spectra datasets (Luck et al. 2016, 2015).

Metabolic profiling of patients affected by monogenic inborn errors of metabolism is a particularly useful approach to address diagnosis/prognostic issues, and to provide insights into the pathophysiology of the disease (Emwas et al. 2015; Mussap et al. 2013; Nicholson et al. 2012). Recently, a proof of concept of the relevance of using ^1^H-NMR-based metabolic profiling to explain the recurrence of Acute Intermittent Porphyria (AIP) attacks was done (Carichon et al. 2014). Porphyrias are a group of eight metabolic disorders of the heme biosynthesis pathway resulting in the accumulation of specific heme and porphyrin precursors. The most frequent of them are AIP and Porphyria Cutanea Tarda (PCT). AIP, which is caused by a deficiency in the enzymatic activity of hydroxymethylbilane synthase (HMBS), is characterized by acute attacks, typically consisting of severe abdominal pain, nausea, constipation, confusion and seizure. Notably, between attacks, patients are asymptomatic. PCT, which is caused by an enzymatic deficiency of uroporphyrinogen III decarboxylase, is characterized by skin fragility and blisters, and is the most frequent type of porphyria worldwide. A sporadic subtype (sPCT, 75% of cases) is most often identified in male patients with hepatic disease, whereas a familial subtype (fPCT, 25% of cases) is transmitted as an autosomal dominant mendelian disorder of low penetrance (Puy et al. 2010).

In the present study, we performed the urinary metabolic profiling of patients with asymptomatic AIP (aAIP), sPCT and fPCT to determine if the urinary metabolome of aAIP can yield information on the mechanism of the disease, besides the identification of porphyrin precursors, and can fuel new pathophysiological hypotheses, and to compare the performances of the rule-mining based algorithm with Partial Least Square Discriminant Analysis (PLS-DA).

## Results

### Local rule-mining based approach on metabolomic data

We first tested whether metabolic profiling could help to discriminate sPCT from fPCT patients, but we could not generate significant models and consequently the information was not clinically relevant. Consequently, sPCT and fPCT patients where merged together (s/fPCT patients).

**Fig. 1:**
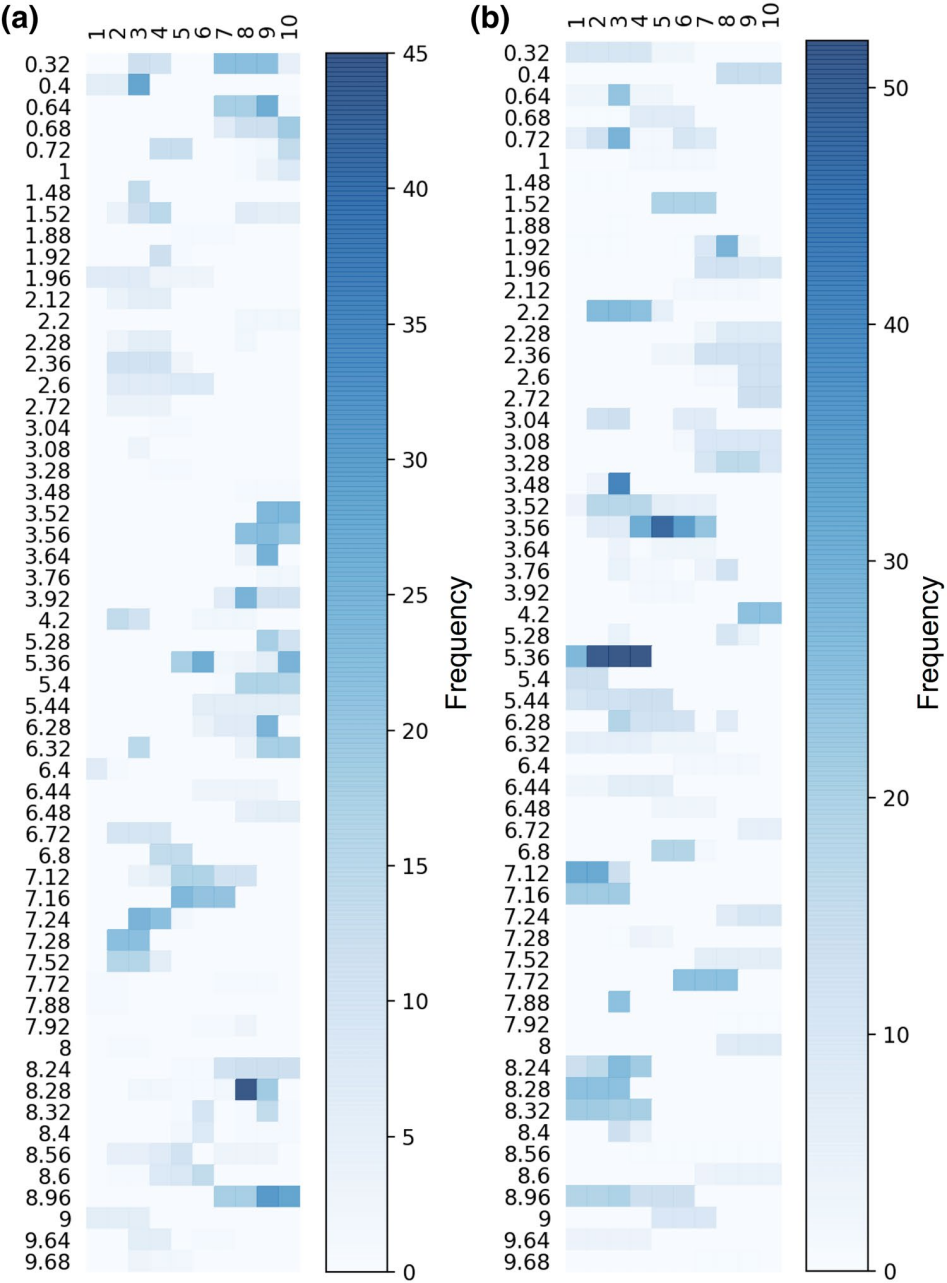
Representation of the 1D rules for the discovery cohort dataset. Each horizontal segment corresponds to a 1D rule characterized by its variable condition: the bucket’s name and the set of covered bins. We only show the rules corresponding to buckets for which rules could be generated for the two classes (i.e., aAIP and s/fPCT). The color scale reflects the frequency of the buckets values covered by the rules over the leave-one-out splits. The more robust the rule, the darker it will be. On the figures on the left **a**, the rules corresponding to the s/fPCT class and on the right figures, the rules corresponding to the aAIP class **b**

We then compared ^1^H-NMR urinary spectra of aAIP with those from s/fPCT patients. For the rule-mining based model, 80 buckets out of the 210 were present at least once in the relevant candidate 1D rules highlighted. 57 of the variables on which rules were highlighted indicate that the same variable (a “bucket”) can be found in 2 different rules (Fig. 1) and that a single molecule (as defined by buckets) can belong to both groups of diseases, but that this is the range of concentrations that is discriminatory. As an example, buckets 2.6 and 2.72, which represent citrate, are found in high concentration in urines of aAIP individuals compared with a far lower concentration in urines of individuals with s/fPCT (and without overlap). We evaluated the relevance of these rules in the classification step by applying a logistic regression on the rules (previously transformed in binary variables). The local models had good and statistically significant performance on the discovery cohort with a F1 score of 0.62 (p value of 0.03).

**Fig. 2:**
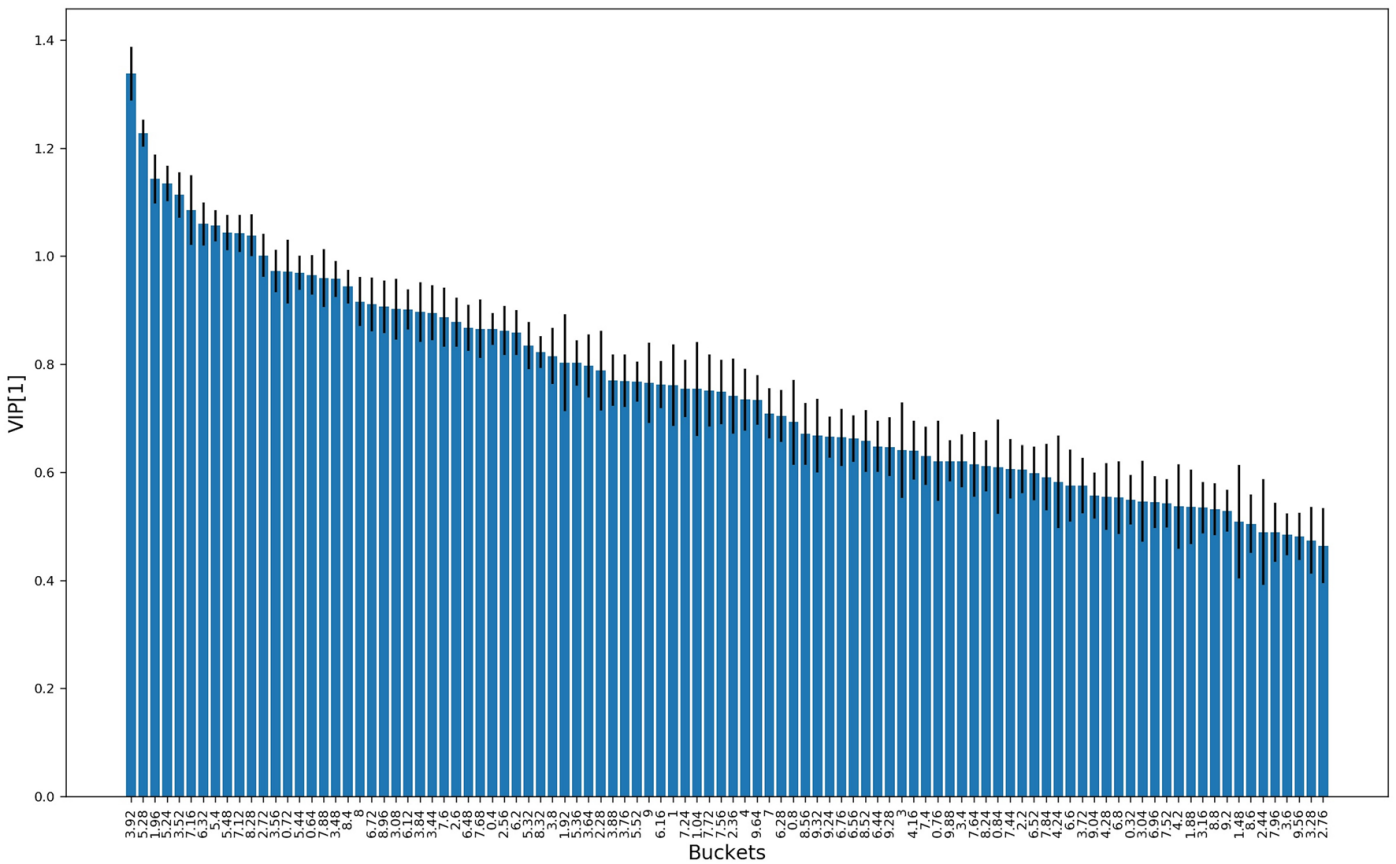
Variables of influence in the projection of the first component (VIP[1]) of the PLS-DA. The bar corresponds to the normalized mean weights of the most discriminative variables in the projection of the first component of the PLS-DA models. The standard errors of the weights are indicated on the figure

To further validate our findings, and to test the consistency of the rule-mining based method, we run a PLS-DA models on the discovery cohort. The PLS-DA model provided a F1 score of 0.63 (p value of 0.003). The Variables of Influence in the Projection (VIPs) of the first component of the PLS-DA model are shown in the Fig. 2. In line with the consistency between the two methods, the VIPs in the PLS-DA models were almost the same found with the rule-mining based method. Together, these results indicate that urinary metabolic profiling can discriminate individuals with aAIP from those suffering with sPCT and fPCT. The local model has an enhanced explanatory power providing more meaningful and easily interpreted information than that provided by a classical model such as PLS-DA. However, it seems that the explanatory power comes at the cost of a small decrease in performance. These findings raise the issue of the pathophysiological rationale supporting these metabolomic profiles.

### Differences in metabolomic profiles of aAIP and PCT patients

We next investigated the nature of the metabolites selected by the local model to identify the urine metabolomic profiles predictive of aAIP and PCT. Among the buckets identified with the rule-mining algorithm, some of them corresponded to metabolites involved in glucose metabolism: acetate (1.92, 1.96), citrate (2.6, 2.72), pyruvate (2.36), glucose (3.48, 3.52, 3.56, 3.76, 3.92, 5.28), and glycogen (3.64, 5.36, 5.4, 5.44), indicating that glucose metabolism pathways are likely impacted by hepatic porphyrias. The corresponding metabolites were identified manually using the Human Metabolome Database (HMDB, http://www.hmdb.ca/). Notably, some rules characterized by the same bucket for both aAIP and PCT patients were disjoint (i.e., covered disjoint sub-regions of the bucket space) (Fig. 1). The disjoint rules indicate that aAIP patients were specifically characterized by higher concentrations of acetate, citrate and pyruvate, the inverse being observed for PCT patients. These findings illustrate that our rule-mining algorithm allows the classification of groups of patients based on the local trends (i.e., range of values) of some variables.

We estimated the absolute concentration of some metabolites identified during metabolic profiling (acetate, pyruvate and citrate) in the discovery and validation cohort by comparing the peak integrals of the corresponding buckets obtained from the ^1^H-NMR spectrum to the residual solvent peak for which the concentration is known (1 mM of TSP—Trimethyl silyl propionate of sodium salt as the ^1^H-NMR chemical shift reference) in the 2014 cohorts of aAIP (n = 31) and PCT (n = 21) patients (those used to build the models) and also in the 2015 replicative cohort which was made of 21 PCT patients. aAIP patients were specifically characterized by relatively higher concentrations of acetate (p value = 0.0004, one-way ANOVA), pyruvate (p value < 0.0001, one-way ANOVA) and citrate (p value < 0.0001, one-way ANOVA) (Table 1) compared with patients with s/fPCT. Notably, the concentrations of these metabolites were within the reference range (based on the urine concentrations collected in the HMDB database). There was no significant difference in the concentrations of acetate, pyruvate and citrate between sPCT and fPCT. Together, these results indicate that the urines of aAIP individuals are characterized by a significant increase of concentrations of glycolytic intermediates (citrate, acetate and pyruvate) compared with urines of patients with PCT.

**Table 1:** Concentrations of the identified buckets

	aAIP 2014	PCT 2014	PCT 2015	Reference	*p* value
Acetate b. 1.96 (3H)	0.015 ± 0.014	0.0046 ± 0.003	0.006 ± 0.007	0.0025–0.106	0.0004
Pyruvate b. 2.36 (3H)	0.0061 ± 0.0017	0.003 ± 0.0013	0.004 ± 0.0013	0.018–0.104	< 0.0001
Citrate b. 2.56 + b. 2.6 (2H)	0.03 ± 0.007	0.02 ± 0.003	0.01 ± 0.0046	0.046–0.484	< 0.0001

The mean ± standard deviation of the concentration of the identified buckets are given in mmol/mmol urinary creatinine for each groups of patients (aAIP and PCT) for the discovery (2014) and the validation (2015) cohort

## Discussion

We provide evidence that a model derived from a data mining approach based on rule generation combined with logistic regression is an innovative and straightforward tool for metabolomic profiling. Local models generate easily interpretable rules compared to the classical PLS-DA method. In addition, these models provide information on the distribution of the groups of diseases with respect to range of the spectral values. This point is critical as it can be possible to translate this range of spectral values into of metabolite concentrations. In turn, the validation of the biological relevance of the potential biomarker identified by these models can immediately be done by measuring its concentration in independent datasets.

Our approach might provide critical information on the potent metabolic disturbances associated with porphyrias. Metabolic profiling could not help to discriminate sPCT and fPCT patients. Indeed, we could not generate significant models and consequently the information was not clinically relevant but strongly suggests that despite different molecular basis, these disorders present with a similar urinary metabolic profile suggesting a close relationship in their pathophysiology. Conducting a future study with more individuals might help to generate more significant models.

The fact that the majority of the discriminating variables were metabolites implicated in glycolysis and that urines of aAIP have relatively higher concentrations of acetate, citrate and pyruvate compared with PCT patients suggest that metabolic reprogramming operates during aAIP, even in the absence of attacks. The biological rationale that supports such changes remain to be determined but it is tempting to speculate that the biochemical consequences of HMBS activity could promote, directly or indirectly, alterations in glycolytic process that occur in the mitochondria. These results must be considered in the perspective of the relationships that occur between heme synthesis and mitochondrial energetic metabolism. Indeed, the heme biosynthesis pathway is closely linked to the tricarboxylic acid (TCA) cycle that provides succinyl-CoA as a carbon source for the initial aminolevulinic acid (ALA) synthesis step (mediated by ALA synthase) of heme biosynthesis. Defects in mitochondrial energetic metabolism occur during active AIP (Homedan et al. 2014), and TCA seems to be profoundly affected at the level of enzymes that orients the first step of the TCA flux and the synthesis or utilization of succinyl-CoA. The increase of aminolevulinate synthase 1 (ALAS1) activity during AIP probably consumes most of the succinyl-CoA available in the mitochondria to support the transfer of succinyl-CoA from the TCA cycle to ALA synthesis. Hence, cataplerosis likely occurs in such a way that the TCA cycle is unable to provide reduced substrates to the respiratory complexes.

Whether the detection of alterations of TCA cycle in aAIP urines, considering that they reflect mitochondrial energetic disturbances, reveals systemic (produced by the liver) or local disturbances (i.e., in the tubular epithelium of the kidney) remains an unresolved issue. However, the kidney is the third organ involved in heme biosynthesis (5% of the heme is synthetized by the kidney) and perturbations in heme synthesis affect renal homeostasis. Our findings also suggest for the first time that metabolic reprogramming associated with AIP occurs even in the absence of overt symptoms or attacks, and support the hypothesis that aAIP can evolve as a smoldering disease with long-term deleterious consequences. In addition, the identification of disturbances of glycolysis in aAIP patients could foster therapeutic strategies targeting ALAS1 activity and limiting the cataplerosis of TCA cycle, which therefore could also be used in asymptomatic carriers.

In conclusion, we provide evidence that a rule-mining algorithm is a useful and straightforward method to select variables, and to generate easily interpretable sets of variables and ranges of variable values that define subpopulations with a high risk for the outcome of interest. Using ^1^H-NMR spectroscopy-based metabolic profiling of individuals with porphyria, we demonstrate that urines of aAIP patients are enriched in glycolytic intermediates that could reflect disturbances of glycolytic intermediates throughout the TCA cycle in relation to mitochondrial metabolic associated with a decrease in HMBS activity.

## Materials and methods

### Patients

Between 2014 and 2015, 73 individuals with normal renal function and with AIP or PCT who were diagnosed and followed at the French Porphyria Center (Centre National Maladies Rares Porphyries, http://www.porphyrie.net/) were included in the study. The criteria for AIP, sPCT and fPCT diagnosis followed the European Porphyria Network guidelines (Anderson et al. 2005; Bonkovsky et al. 1991). The asymptomatic carrier status of AIP, “aAIP” (i.e., individuals included in this study), is defined after a family screening to identify individuals with latent disease and is based on a deficient HMBS enzymatic activity complemented with a DNA analysis by direct sequencing to identify the causative mutation in the HMBS gene (which requires prior identification of the mutation in a related affected family member). PCT is characterized by fragile skin, bullae, hypertrichosis, pigmentation that may be acquired (sporadic PCT, “sPCT”) or inherited (familial PCT, “fPCT”). Most patients with sPCT have chronic underlying liver cell damage with iron overload related to alcohol, C hepatitis, or haemochromatosis. The diagnosis of PCT is confirmed by the detection of specific porphyrin profile in urines, feces and plasma. The differential diagnosis between sPCT and fPCT was realized by measuring uroporphyrinogen III decarboxylase activity in erythrocytes and by UROD gene analysis.

For each patient, demographic and clinical information was retrieved. Urines of aAIP individuals were collected at annual systematic follow-up, and urines from PCT individuals at the time of the diagnosis. Urine samples were stored at − 80 °C until metabolomic analyses. The 2014 cohort corresponds to the discovery cohort (52 patients). Two groups of patients were defined for our analysis corresponding to 31 patients with aAIP, and 21 with PCT, including 14 patients with sPCT, 7 patients with fPCT. These two porphyrias groups (aAIP and PCT) correspond to the main target variables. The 2015 cohort contains 21 PCT individuals (14 and 7 patients with sPCT and fPCT respectively) and was used as an independent validation replicative cohort for the measurement of candidate metabolites. The characteristics of patients in these two cohorts are provided in Table 2.

**Table 2:** Patients characteristics

	Normal	fPCT (n = 14)	sPCT (n = 28)	aAIP (n = 31)	*p* value
Age (years)		52.3 ± 3.7	55.8 ± 2.4	59.3 ± 2.7	0.3
Sexe ratio (female)		37%	37%	58%	0.1
ALA	< 38 µmol/L	28.5 ± 12.8	31.3 ± 7.7	36.6 ± 7.3	0.8
PBG	< 9 µmol/L	2.3 ± 7.8	4.2 ± 4.4	23.9 ± 4.2	0.003
Total porphyrins	< 250 nmol/L	7375 ± 1616	6277 ± 967	855 ± 1024	0.002
Coproporphyrin III	40–60%	1.9 ± 1.1	4 ± 0.6	–	0.11
Coproporphyrin I	20–30%	2.1 ± 3.4	8 ± 1.9	–	0.14
Pentacarboxyporphyrin	N: <3%	5 ± 1.8	7 ± 1	–	0.33
Hexacarboxyporphyrin	N: <2%	1.4 ± 0.3	1 ± 0.1	–	0.28
Heptacarboxyporphyrin	N: <3%	36.6 ± 3	33.1 ± 1.7	–	0.3
Uroporphyrin	N: 10–15%	58 ± 24	44 ± 14	–	0.05

Continuous variables are expressed as mean ± standard deviation; categorical variables are expressed as percentage (%) of the total number of patients (n)

### ^1^H-NMR spectroscopy

The urine samples were prepared with chemical products from Sigma (Sigma Aldrich, Saint Quentin Fallavier, France) to obtain a final volume of 600 μL (400 μL of urine; 160 μL of 200 mM phosphate buffer at pH 7.4, 1 mM of TSP—Trimethyl silyl propionate of sodium salt as the NMR chemical shift reference-, 6 mM of NaN3; 40 μL D2O). Urine ^1^H-NMR spectra were measured at 300K on a Brucker Avance II 500 MHz spectrometer (Brucker Biospin GmbH, Rheinstetten, Germany) equipped with a SampleXpress automation sample changer and a standard 5 mm BBI probe with Z-gradient. The spectra acquisition was based on a 1D Nuclear Overhauser Effect Spectroscopy (NOESY) pulse sequence with pre-saturation for water suppression. The parameters used for the pulse sequence were as follows: a relaxation delay of 1 s, a mixing time of 100 ms, an acquisition time of 1.36 s and a 90-degree pulse length of 8 μs. Data points (32K) were collected during 64 scans with a spectral width of 20 ppm. The preprocessing of the urine ^1^H-NMR spectra was performed with MestReNova 8.0 software. A line-broadening factor of 0.3 Hz prior to Fourier transformation was applied. The spectra were then phased, baseline corrected, and referenced to TSP. Each ^1^H-NMR spectrum was reduced by an equidistant binning method from 0.24 to 10.00 ppm with a bin width of 0.04 ppm to limit misalignment problems. The spectral regions corresponding to urea (5.52–6.04 ppm) and water (4.28–5.24 ppm) were deleted to remove variability because of the suppression of water. Then, as mean of normalization and prerequisite to our method we discretized the 210 remaining buckets with a 10 quantile-based binning for the main analysis (i.e., aAIP vs. PCT) and with a 5 quantile-based binning for the secondary analysis (i.e., sPCT vs. fPCT). Two NMR spectra datasets were obtained from the discovery cohort: one with 52 individuals and 210 buckets for main analysis (i.e., aAIP vs. PCT) and one with 21 individuals and 210 buckets for the secondary analysis (i.e., sPCT vs. fPCT). One NMR spectra dataset was obtained from the validation cohort containing 21 individuals and 210 buckets for the secondary analysis (i.e., sPCT vs. fPCT). Finally, the resulting matrices were centered and scaled to unit variance for the PLS-DA. The dataset before normalization containing the two cohorts is available in supplementary information (S1 Dataset).

### Supervised analysis

For the supervised analysis, we used a rule-miming based approach that combines a supervised variable selection step and a classification step (Luck et al. 2016). The rationale for the use of this approach is to avoid overfitting and provide more precise information compared to conventional methods. These approaches were performed on the discovery cohort for the prediction of the different type of porphyria.

#### Variable selection

The variable selection step is based on the combination of two univariate approaches: a global one, based on the χ^2^ test (Scikit-learn project implementation (Pedregosa et al. 2011)), and a local one, based on a supervised rule mining algorithm (in-house implementation) (Luck et al. 2015, 2016). The global approach first selected variables with a chi2 test significant p value < 0.05 leading to a subset of variables. We did not perform a Bonferroni correction because there is a high probability that the test is substantially conservative because the Bonferroni method would require p values to be smaller than 0.05/210 to declare significance since adjacent buckets tend to be highly correlated. Secondly, the further local feature selection step must perform a correction.

This set of selected variables generated by the global approach was next used to perform the local approach, which is based on a rule-mining algorithm that locally and exhaustively explores the variable space (see Algorithm 1). The objective is to highlight local phenomena characterized by subgroups of subjects. These relationships are under the form of 1-dimensional rules (1D rules) where variable condition corresponds to a range of values of a given variable observed in the dataset. To identify the most discriminative rules, we used as a rule quality measure the z-score and the rule modality size. The z-score tests whether the proportions of subjects with the target modality in the rule and in the entire dataset are different. We selected rules with a z-score > 1.96 corresponding to a confidence level of 95%. The rule modality size is the number of subjects in the rule having one of the two modalities of interest. Taking into account the few number of subject in the different cohort, we selected rules with a rule modality size > 5 to ensure robust and generalizable rules.

**Algorithm 1:**
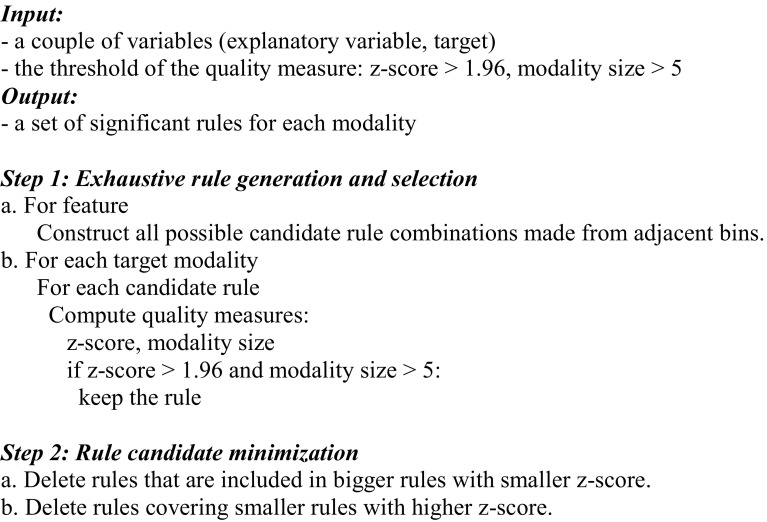
Supervised rule mining

#### Classification

For the classification step, we learned a classical L2 penalized (C parameter fixed to 1) logistic regression (L2-LR) on the rules [Scikit-learn project implementation (Pedregosa et al. 2011)]. The rules were represented under the form of local binary variables of the length of the number of subjects. These local binary variables are encoded as follows: 1 means that the subject belongs to the rule and 0 indicates the subject is not in the rule (Luck et al. 2015, 2016).

#### Evaluation and comparison of our approach with PLS-DA

The predictive performance was evaluated using a leave-one-out cross validation and the F1 score with a permutation test of 2000 runs. A p value < 0.05 was considered to be significant. As a means of another validation and in order to prove the consistency of the results found with our methods, we run a PLS-DA on the same dataset as for the rule-mining based approach [Scikit-learn project implementation (Pedregosa et al. 2011)]. For the PLS-DA, the evaluation procedure was the same as for the rule-mining approach (i.e., the predictive performance of the models was evaluated using the F1-score with a permutation test of 2000 runs). A p value below 0.05 was considered to be significant.

#### Biomarkers validation and quantification

A simple estimate of absolute concentration of potential biomarkers in the discovery and validation cohort has been obtained from the ^1^H-NMR spectrum by comparing the peak integrals of the corresponding buckets to the peak for which the concentration is known (1 mM of TSP—Trimethyl silyl propionate of sodium salt as the ^1^H-NMR chemical shift reference-) (Corol et al. 2016; Jauhiainen et al. 2014), and using the profiler of Chenomx NMR Suite 7.1. The mean concentrations per group of patients (aAIP, sPCT and fPCT) were compared using a one-way analysis of variance test (ANOVA). A p value < 0.05 was considered to be significant.

## Electronic supplementary material

Below is the link to the electronic supplementary material.

Supplementary material 1 (CSV 148 KB)
