# Correction to: Urinary metabolic profiling of asymptomatic acute intermittent porphyria using a rule-mining-based algorithm

**DOI:** 10.1007/s11306-018-1320-5

**Published:** 2018-01-30

**Authors:** Margaux Luck, Caroline Schmitt, Neila Talbi, Laurent Gouya, Cédric Caradeuc, Hervé Puy, Gildas Bertho, Nicolas Pallet

**Affiliations:** 1 INSERM U1147, Centre Universitaire des Saints Pères, Paris, France; 2 Université Paris Descartes, Paris, France; 3 Sorbonne Paris Cité, Paris, France; 4 Institut Hypercube, Paris, France; 5 Centre Francais des Porphyries, Hôpital Louis Mourier, Assistance Publique-Hôpitaux de Paris, Colombes, France; 6 INSERM U1149, CNRS ERL 8252, Center for Research on Inflammation (CRI), Université Paris Diderot, Site Bichat, Sorbonne Paris Cité, Paris, France; 7 Laboratory of Excellence, GR-Ex, Paris, France; 8 UMRS 8601 CNRS, Paris, France; 9 Service de Biochimie, Hôpital Européen Georges Pompidou, Assistance Publique-Hôpitaux de Paris, 20, rue Leblanc, 75015 Paris, France

## Correction to: Metabolomics (2018) 14:10 10.1007/s11306-017-1305-9

The article Urinary metabolic profiling of asymptomatic acute intermittent porphyria using a rule-mining-based algorithm, written by Margaux Luck, Caroline Schmitt, Neila Talbi, Laurent Gouya, Cédric Caradeuc, Hervé Puy, Gildas Bertho and Nicolas Pallet was originally published Online First without open access. After publication in volume [14], issue [1], Citation ID[10] the author decided to opt for Open Choice and to make the article an open access publication. Therefore, the copyright of the article has been changed to © The Author(s) 2018 and the article is forthwith distributed under the terms of the Creative Commons Attribution 4.0 International License (http://creativecommons.org/licenses/by/4.0/), which permits use, duplication, adaptation, distribution and reproduction in any medium or format, as long as you give appropriate credit to the original author(s) and the source, provide a link to the Creative Commons license and indicate if changes were made. The original article has been corrected.

